# Antitumor efficacy of silver nanoparticles reduced with β-D-glucose as neoadjuvant therapy to prevent tumor relapse in a mouse model of breast cancer

**DOI:** 10.3389/fphar.2023.1332439

**Published:** 2024-01-25

**Authors:** Moisés Armides Franco Molina, David Reding Hernández, Paola Leonor García Coronado, Jorge R. Kawas, Diana G. Zárate Triviño, Sara Paola Hernández Martínez, Beatriz Elena Castro Valenzuela, Cristina Rodríguez Padilla

**Affiliations:** ^1^ Laboratorio de Inmunología y Virología, Facultad de Ciencias Biológicas, Universidad Autónoma de Nuevo León, San Nicolás de los Garza, Nuevo León, Mexico; ^2^ Posgrado Conjunto Agronomía-Veterinaria, Universidad Autónoma de Nuevo León, General Escobedo, Nuevo León, Mexico; ^3^ Facultad de Agronomía, Universidad Autónoma de Nuevo León, General Escobedo, Nuevo León, Mexico

**Keywords:** neoadjuvant, tumor relapse and recurrence, silver nanoparticles, breast cancer, breast cancer relapse

## Abstract

**Introduction:** Neoadjuvant therapy constitutes a valuable modality for diminishing tumor volume prior to surgical resection. Nonetheless, its application encounters limitations in the context of recurrent tumors, which manifest resistance to conventional treatments. Silver nanoparticles (AgNPs) have emerged as a promising alternative for cancer treatment owing to their cytotoxic effects.

**Methods:** Cellular viability was assessed by Alamar blue assay in 4T1 breast cancer cell line. Silver biodistribution was detected by an inductively coupled plasma optical emission spectrometer in an *in vivo* mice model. For neoadjuvant evaluation, mice were randomized and treated intratumoral with AgNPs-G or intraperitoneally with doxorubicin (DOX) as a control. Recurrence was determined after 170 days by counting lung metastatic nodules (dyed with Bouin solution) with histological confirmation by H&E. Masson’s stain, Ki67 immunohistochemistry, and a TUNEL assay were performed in lungs from treated mice.

**Results:** AgNPs-G reduced 4T1 cell viability and in an *ex vivo* assay the AgNPs-G decreased the tumor cell viability. After intravenous administration of AgNPs-G were detected in different organs. After intratumor administration, AgNPs-G are retained. The AgNPs-G treatment significantly reduced tumor volume before its surgical resection. AgNPs-G reduced the development of lung metastatic nodules and the expression of Ki67. TUNEL assay indicated that AgNPs-G didn’t induce apoptosis.

**Conclusions:** We concluded that intratumor administration of AgNPs-G reduced tumor volume before surgical resection, alongside a reduction in lung metastatic nodules, and Ki67 expression. These findings provide valuable insights into the AgNPs-G potential for intratumor and neoadjuvant cancer therapies. However, further research is needed to explore their full potential and optimize their use in clinical settings.

## 1 Introduction

Cancer is one of the major health problems worldwide. Approximately 609,820 individuals are projected to succumb due to cancer in the United States by the end of 2023, which is equivalent to 1,670 deaths daily. Among all cancers, breast cancer is highly prevalent in women; one in eight women experiences breast cancer during her life, and it is considered the primary cause of woman mortality worldwide (Siegel et al., 2023; Mohammadpour et al., 2022).

The use of neoadjuvant therapy, in conjunction with traditional anti-cancer therapies, such as chemotherapy, has been regarded as an early intervention strategy for patients with breast cancer, particularly those presenting locally advanced tumors. This approach offers the advantage of downsizing both the tumor and regional metastatic lymph nodes, thereby enhancing surgical outcomes through an increased likelihood of breast-conserving surgery (Abdel-Raqez et al., 2022).

Breast cancer cells have the capacity to disseminate to distant organs, specifically the lungs, liver, bone, and brain. In triple-negative breast cancer (TNBC), the incidence of lung metastasis can be as high as 40% when compared to 20% in non-TNBC. Approximately 60% of metastatic breast cancer patients experience lung or bone metastasis during their lifetime. Unfortunately, the prognosis remains grim for breast cancer patients with lung metastasis, despite undergoing chemotherapy, targeted therapy, and endocrine therapy tailored to molecular receptor profiles; the median survival after treatment for lung metastasis is only 22 months, emphasizing the challenges in improving life expectancy in this context (Jin et al., 2018).

Persistent cancer cells result from the undetectable residual cancer cells in the body post-treatment, commonly after surgical procedure, that can subsequently and unpredictably lead to metastatic relapses. The occurrence of residual disease after the initial excisional breast cancer biopsy has been documented to vary between 45% and 70% of patients. A bad surgical margin is linked to a heightened risk of local recurrence, which serves as the primary rationale for considering re-excision in patients whose margin status is questionable or deemed inadequate (Chae, et al., 2013). Despite most patients with metastatic cancers initially achieving what oncologists describe as a partial or complete response to cancer drug therapies, relapses inevitably occur. These relapses may manifest months or years later, even in the absence of discernible tumors. This persistence of residual disease establishes a reservoir that fosters the development of drug-resistant cells (Shensi, et al., 2020).

To overcome these challenges, nanotechnology has proven to be a useful tool against cancer cells. Materials measuring 300 nm in size can be internalized by individual cells, whereas those with dimensions below 70 nm can even penetrate cellular nuclei, potentially leading to significant harm. Among nanomaterials, silver nanoparticles (AgNPs) have proven to be a useful tool against cancer due to their physicochemical characteristics such as small size of less than 100 nm, high specific surface area, and remarkable reactivity. Silver (Ag) and AgNPs have recently garnered attention as potential agents for cancer treatment due to their ability to cause cytotoxic effects induced by the release of ions when is lysosomal entrapment, and generate oxidative stress, deterioration of the cell membrane followed by cell cycle arrest, inflammatory responses, DNA damage, and apoptosis (Beyene et al., 2017; Tao et al., 2021), through different signal pathways (Kovács et al., 2022). Furthermore, our prior research has established that beta-D-glucose-reduced silver nanoparticles (AgNPs-G) exhibit significant cytotoxicity against several breast cancer cell lines (MCF7, MDA-MB-231, SK-BR3, and murine 4T1); however, vaccination with 4T1 breast cancer cells treated with AgNPs-G failed to prevent tumor establishment (Felix-Piña et al., 2023). Notably, the antitumor effect has not yet been examined. Consequently, the primary objective of this study was to evaluate the effectiveness of AgNPs-G as a neoadjuvant therapy in preventing tumor relapse in a mouse model of breast cancer.

However, due to the cytotoxicity properties mentioned by our AgNPs-G, there is pressing need for more efficient and safer neoadjuvant treatments in the realm of breast cancer management. The aim of this study was to assess the efficacy of AgNPs-G previously synthetized and characterized by our research group (Felix et al., 2023) as a neoadjuvant therapy in tumor relapse using the 4T1 mouse model of breast cancer that exhibit similar characteristics to TNBC (Fantozzi and Christofori, 2006).

## 2 Materials and methods

### 2.1 Silver nanoparticles reduced with β-D-glucose (AgNPs-G) synthesis

The AgNPs-G were synthesized and characterized by our research team as previously described by us (Felix et al., 2023). In brief, 10 mL of aqueous solution of β-D-glucose at a concentration of 0.3 M in a beaker was exposed to a water bath in glycerol at 120°C for 5 min. Subsequently, 100 μL of AgNO_3_ solution was added dropwise. A total of 2.5 mM and 10 μL of 0.1 M NaOH solution were added until the color changed to yellow, which is indicative of AgNPs-G formation. In all preparations, deionized water provided by a comprehensive Milli-Q water purification solution system was used (Merck Millipore, Billerica, MA, United States).

### 2.2 Cytotoxic effect

4T1 cells were seeded at a density of 5 × 10^3^ per well in a 96-well microplate with 200 µL of Dulbecco’s modified Eagle’s medium (DMEM) supplemented with antibiotics and 5% FBS. The cells were incubated overnight at 37°C in an atmosphere of 95% air and 5% CO_2_. Then, DMEM was removed and replaced with the AgNPs-G treatment diluted in DMEM (0, 5.4, 6.75, 8.1, 9.45, 10.8, 12.3, 13.5, and 16.2 μg/mL) and incubated for 24 h with conditions previously mentioned. After that, the medium was removed, and the wells were washed with 1x phosphate-buffered saline (PBS). Then, 100 µL of Alamar Blue (Sigma, St. Louis, MO, United States) at 20% *v/v* were added, and the mixture was incubated for 4 h with conditions previously described. The fluorescence readings were performed using a Synergy HT™ spectrophotometer at an excitation wavelength of 535 nm and an emission wavelength of 590 nm. The percentage of cytotoxicity was defined using the following equation:

$$\%\;Cytotoxity=100-\left(\left(\frac{A}{B}\right)x100\right).$$

where A represents the average absorbance of cells with treatment and B represents the average absorbance of cells that did not receive treatment.

### 2.3 Animals and tumor induction

Female BALB/c mice (6–8 weeks old) were acquired from Bioterium and housed under alternating dark and light cycles for 12 h each and maintained on food and water *ad libitum.* Tumors were generated after subcutaneous (s.c.) injection of 1 × 10^6^ 4T1 cells in the dorsal area of mice. All animals were treated in accordance with the guidelines with the Committee on the Care and Use of Laboratory Animals of the UANL (CEIBA) and NOM-062-ZOO-1999. For each group, five mice were used for statistical significance.

### 2.4 *Ex vivo* antitumor effect

After 7 days of 4T1 cells inoculated as previously described and only when tumor was palpable, mice were sacrificed, and tumor was resected in sterile conditions. Tumors were cut in fragments of ≈700 mm^3^ and washed in sterile 1X PBS solution. Then, tumor sections were immersed in AgNPs-G treatments diluted in DMEM (8.1 and 16.2 μg/mL) and incubated for 24 h under the conditions previously mentioned. After 24 h of incubation, the medium was removed, and a cell suspension was obtained by perfusion. Alamar Blue assay was performed as previously described.

### 2.5 AgNPs-G biodistribution assay

Biodistribution assay was performed on another group of mice which is different from the previous experiment (10 BALB/c mice). Mice were randomly divided in the following groups: mice bearing tumor which were injected intra-tumorally (i.t.) with AgNPs-G and mice without tumor injected in tail vein with AgNPs-G with 50 mL of 32.4 μg/mL, and physiological saline solution for the control group. Administration was carried out daily for 2 weeks. After that, all the animals were euthanized. After that, the following organs were collected: heart, lungs, skin, liver, kidney, gallbladder, spleen, and tibia, and tumors (from the mice bearing tumor). Organs and tumor were stored at −20°C. Silver detection was carried out in accordance with the AOAC Official Method 2011.14. In brief, organs were grinded followed by digestion with HNO_3_. HCl was added to the digested solution and the mixture was incubated for 20 min. The remaining solution was filtered and diluted in 50 mL of H_2_O. Finally, silver detection was carried out using an inductively coupled plasma optical emission spectrometer.

### 2.6 Neoadjuvant therapy and model of resection

Tumors were induced as previously described. After 2 weeks, mice were euthanized, and tumors were resected in sterile conditions and cut in fragments as previously described. Those fragments (one per mice) were implanted in 25 female BALB/c mice. Mice were anesthetized with ketamine (75 mg/kg)/xylazine (15 mg/kg), and fur in the dorsal area was shaved. Then, a 5-mm-long incision was made in the dorsal area, and tumor fragments were implanted subcutaneously, and the incision was closed with a suture. Then, mice were randomly divided into four groups (control, AgNPs-G with low dose, AgNPs-G with high dose, and doxorubicin). The AgNPs-G low-dose groups received daily i.t. injections of 16.2 μg/mL AgNPs-G, the AgNPs-G high-dose group received daily i.t. injections of 32.4 μg/mL AgNPs-G, the positive control group received a single intraperitoneal injection of doxorubicin (10 mg/kg), and the negative control group received an i.t. injection of saline solution. Injection volumes of AgNPs-G and saline solution were 50 µL. Tumor volume was measured twice a week for 2 weeks using a caliper. The tumor volume was calculated using the formula for an ellipsoid (length × width × height × 0.5236).

Tumors were resected on day 16. For that, mice were anesthetized as previously described and the area around the tumor was shaved using a finisher trimmer. The surgical area was then disinfected using betadine solution followed by a 70% ethanol solution. The primary tumor was then excised by lifting the tumor with forceps and cutting around the base using a surgical blade. Blood vessels feeding the primary tumor were cauterized and the resection site was closed with a suture.

Following primary tumor resection, mice were monitored daily for primary tumor recurrence and morbidity. Any mouse experiencing a primary tumor recurrence or experiencing significant weight loss, obvious distress, or labored breathing was euthanized and the lungs were removed to confirm death due to metastatic disease. Mice surviving more than 170 days after resection were deemed cured.

After monitoring, all surviving mice were euthanized, and the lungs were resected. All lungs were fixed by immersion in Bouin solution overnight for enumeration of tumor nodules under a stereomicroscope.

### 2.7 Hematoxylin and eosin staining

Lung tissues from mice bearing tumor treated with PBS (negative control), doxorubicin (positive control), and AgNPs-G (32.4 μg/mL or 16.2 μg/mL), and fixed, cut, and placed in glass slides were deparaffinized with xylene for 10 min and rehydrated through alcohol gradient (100% alcohol, 95% alcohol, and 70% alcohol for 10 min each). Then, tissues in slides were washed with distilled water. Tissues were stained in hematoxylin for 10 min, rinsed in running warm tap water for 10 min, and then washed in distilled water. Then, contrast was performed in 0.2% eosin up to 45 s. Dehydration of tissues was performed with 95% and 100% ethanol for 10 min each. Slides were mounted with resinous mounting medium.

### 2.8 Masson staining

Lung tissues from mice bearing tumor treated with PBS (negative control), doxorubicin (positive control), and AgNPs-G (32.4 μg/mL or 16.2 μg/mL) fixed in Bouin solution were placed in paraffin blocks, cut, and then placed in glass slides. To perform Masson staining, tissues were deparaffinized with xylene for 10 min and rehydrated through alcohol gradient (100% alcohol, 95% alcohol, and 70% alcohol for 10 min each). Then, tissues were washed in distilled water and rinsed in running tap water for 5–10 min to remove the yellow color. Stain in Weigert’s iron hematoxylin working solution was performed for 10 min and tissues were then rinsed in running warm tap water for 10 min. After one wash step in distilled water, stain in Biebrich scarlet-acid fuchsin solution was performed for 10–15 min and another wash was made. Collagen differentiation was made through staining within phosphomolybdic-phosphotungstic acid solution for 10–15 min (until collagen is not red). After that, aniline blue solution was added to tissues incubating for 5–10 min. Then, tissues were briefly rinsed in distilled water and incubated in 1% acetic acid solution for 2–5 min. Another wash was performed in distilled water. Tissues were dehydrated in 95% ethyl alcohol, absolute ethyl alcohol, and clear in xylene (by immersion), and mounted with resinous mounting medium.

### 2.9 Immunohistochemistry staining

To seek activation of proliferation/death markers in lungs presenting metastatic nodules, IHC was performed. Lung tissues from mice bearing tumor treated with PBS (negative control), doxorubicin (positive control), and AgNPs-G (32.4 μg/mL or 16.2 μg/mL), and fixed, cut, and placed in glass slides were deparaffinized. Then, tissues were rehydrated as previously described in a series of xylenes and ethanol gradient. Antigens were retrieved using citrate pH 6.0 buffer (heated for 45 s) before probing with primary antibodies anti-Ki67 (1:200 dilution, #sc-23900, Santa Cruz). Then, antibodies were detected using Vectastain Elite ABC Universal Kit (#PK-7200) followed by DAB detection and counterstained with hematoxylin. Photographs were taken with a Zeiss microscope coupled to a camera at 10X and 40X.

### 2.10 TUNEL assay

The sample sections were deparaffinized by immersing slides in fresh xylene in a coplin jar for 10 min at room temperature. The slides were then processed according to the manufacturer’s instructions (DeadEnd Fluorometric TUNEL system, #G3250). Images were taken in a fluorescent microscope (Leica DM1000 Fluorescence Microscope).

### 2.11 Statistical analysis

All experiments were carried out in triplicate with a Student’s t-test and an analysis of variance (ANOVA) type experimental study followed by Tukey’s *post hoc* test using GraphPad Prism software (San Diego CA, United States). Letters (a, b, c, and d) show a significant difference (*p* < 0.05) between treatments.

## 3 Results

### 3.1 *In vitro* and *ex vivo* decrease of 4T1 cell viability

**FIGURE 1:**
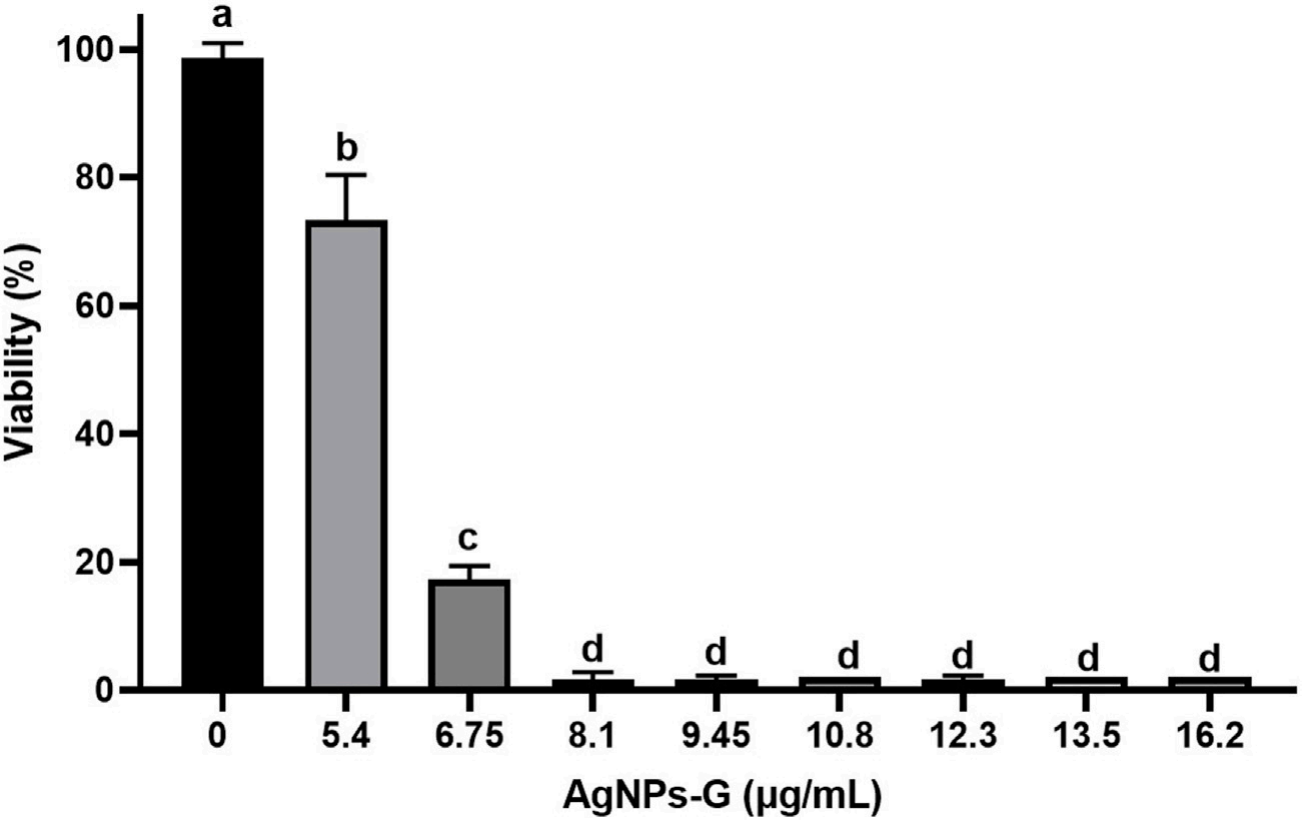
Decrease in cell viability of the 4T1 line induced by AgNPs-G. The cells were treated at different concentrations for a period of 24 h, and subsequently, the viability was determined using the Alamar Blue method. The results were analyzed using an ANOVA test; statistical significance (^a,b,c,d^ *p* < 0.05).

The AgNPs-G treatment decreased the cellular viability of 4T1 breast cancer cell line in all doses tested (5.4–16.2 μg/mL), in a dose-dependent manner (*p* < 0.05) when compared with the control without treatment in a period of 24 h (Figure 1). Starting from the dose of 8.1 μg/mL, cellular viability reached values near to zero.

**FIGURE 2:**
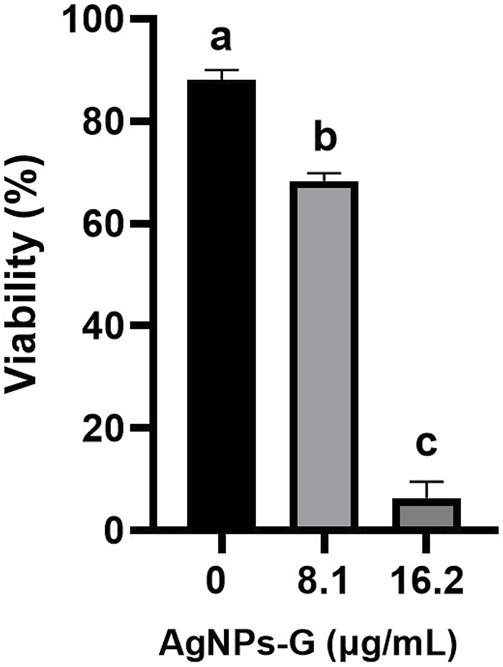
Cytotoxic effect of AgNPs-G against a solid tumor. Tumors formed from 4T1 cells with an approximate volume of 700 mm^3^ were treated with concentrations of 8.1 and 16.2 μg/mL for 24 h. Cells were subsequently obtained by perfusion, and viability was determined by trypan blue. The results were analyzed using an ANOVA test; statistical significance (^a,b,c^ *p* < 0.05).

The lethal doses obtained from *in vitro* experiments of 8.1 and 16.2 μg/mL of AgNPs-G were used against solid tumor in an *ex vivo* experiment, indicating that both doses decreased significantly (*p* < 0.05) the tumoral cells’ viability in a period of 24 h compared with the control without treatment (Figure 2); however, the dose of 16.2 μg/mL exhibit the lowest percentage of cellular viability (4.5% viable tumoral cell).

### 3.2 AgNPs-G intra-tumor accumulation after intravenous administration

**FIGURE 3:**
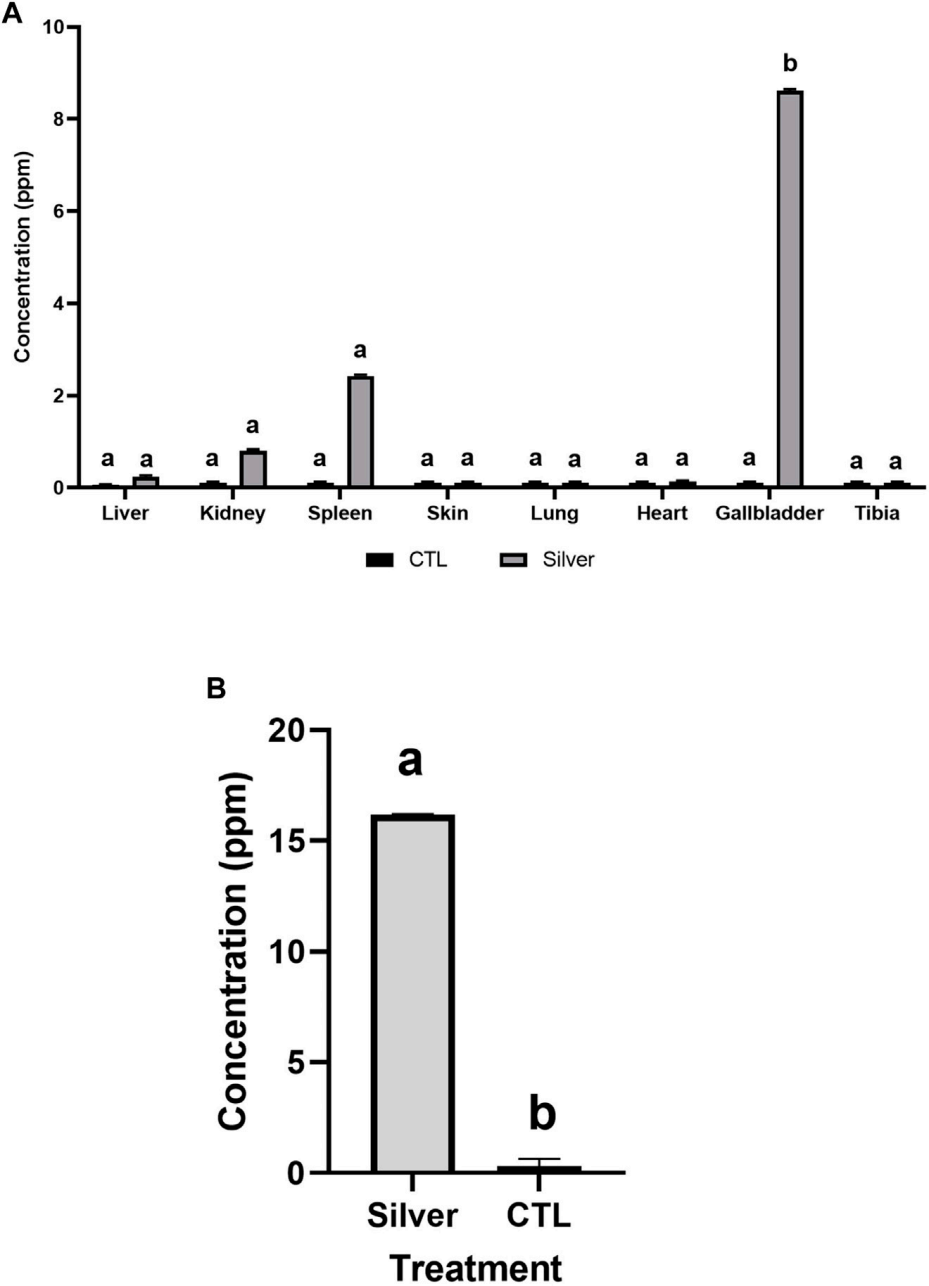
Silver accumulates in different organs when administered i.v. **(A)** but in an i.t., there is a large accumulation of silver in the tumor **(B)**. BALB/c mice were administered with AgNPs-G at a concentration of 32.4 μg/mL over a period of 2 weeks. The results were analyzed using an ANOVA test (*p* < 0.05) for **(A)** and a Student’s t-test (*p* < 0.05) for **(B)**; statistical significance (^a,b^ *p* < 0.05).

After 2 weeks of AgNPs-G administration i.v., the AgNPs-G were detected at different concentrations in the spleen (2.42 ppm), kidney (0.8 ppm), and gallbladder (8.62 ppm); this last organ showed a higher concentration. No silver accumulation was detected in the skin, liver, lung, heart, and tibia (Figure 3A). After 2 weeks of AgNPs-G i.t. administration (Figure 3B), a high concentration of silver was detected in the tumor (16.18 ppm).

### 3.3 Effect of neoadjuvant intra-tumoral treatment with AgNPs-G

**FIGURE 4:**
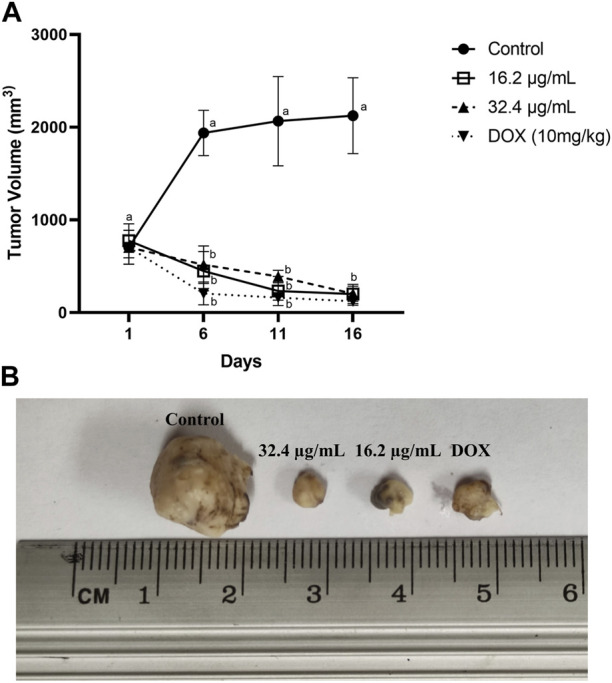
AgNPs-G reduce tumor volume when used in a neoadjuvant manner. Tumor-bearing mice were treated intra-tumorally daily for 16 days (n = 5). **(A)** Tumor volume was measured every 5 days. The control group was only administered physiological solution, and the groups with AgNPs-G consisted of two concentrations of 16.2, 32.4 μg/mL, and DOX. The results were analyzed using an ANOVA test (^a,b^ *p* < 0.05). **(B)** Representative images belong to tumors treated.

After 16 days of daily i.t. administration of AgNPs-G, a significant (*p* < 0.05) decrease in tumor volume was observed at 16.2 μg/mL of AgNPs-G (201 mm^3^), 32.4 μg/mL of AgNp-G (205 mm^3^) doses, and doxorubicin (124 mm^3^) in a time-dependent manner with no significant difference among these groups (*p* < 0.05). We found a higher tumor volume in the control group (2,125 mm^3^) with a significant difference (*p* < 0.05) than that in the treatment groups (AgNPs-G and DOX) (Figure 4). No significant difference (*p* < 0.05) was found in mice weight between groups (Figure 6) during the observation period (170 days).

### 3.4 Tumoral recurrence evaluation

**FIGURE 5:**
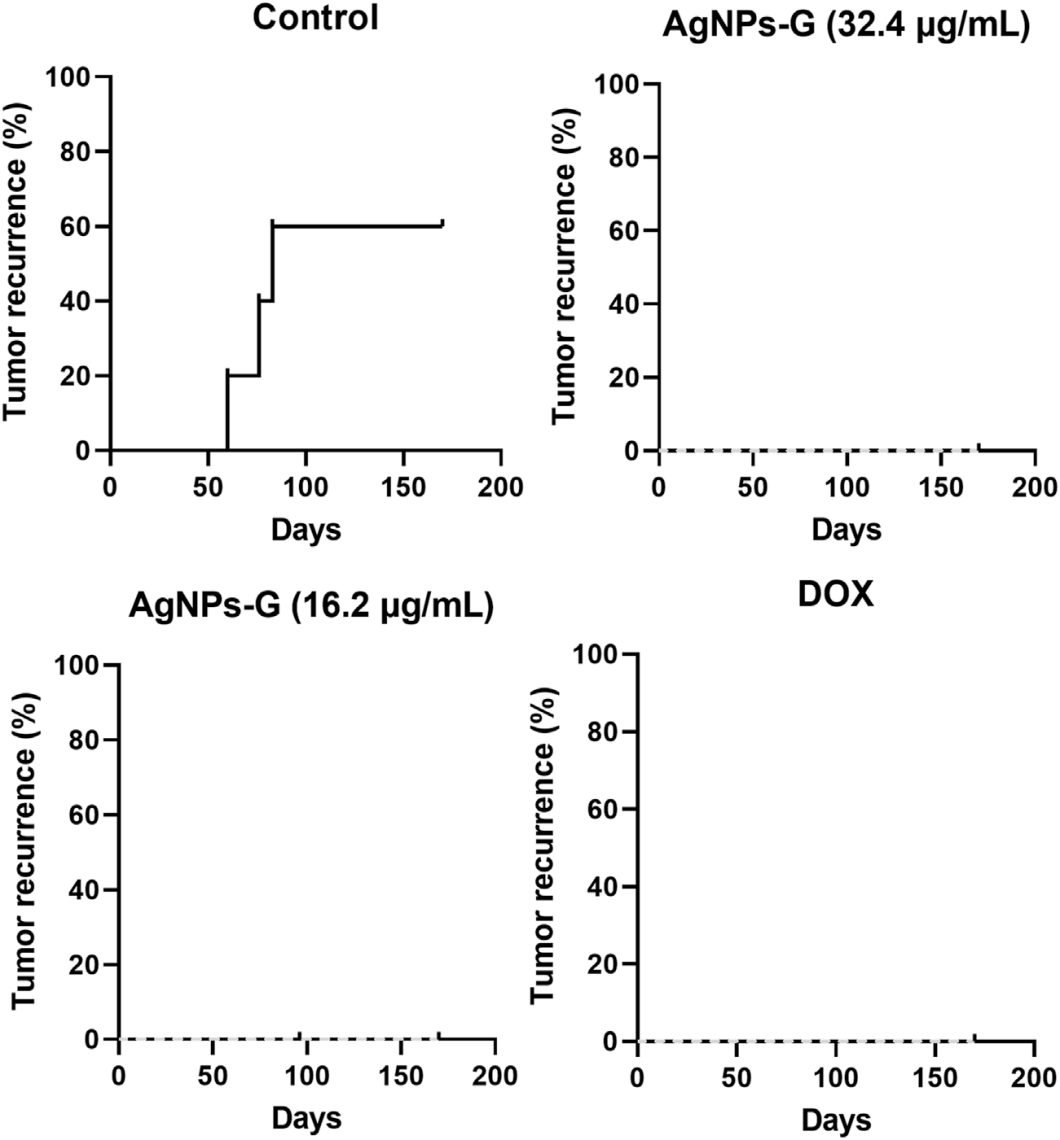
AgNPs-G administered neoadjuvantly reduce recurrence. The development of recurrence in BALB/C mice was followed tumor resection; mice that had breathing difficulties or obvious tumor growth were sacrificed, and the presence of metastasis was confirmed. Recurrence was recorded for 170 days. The dotted line represents the percentage of recurrence.

**FIGURE 6:**
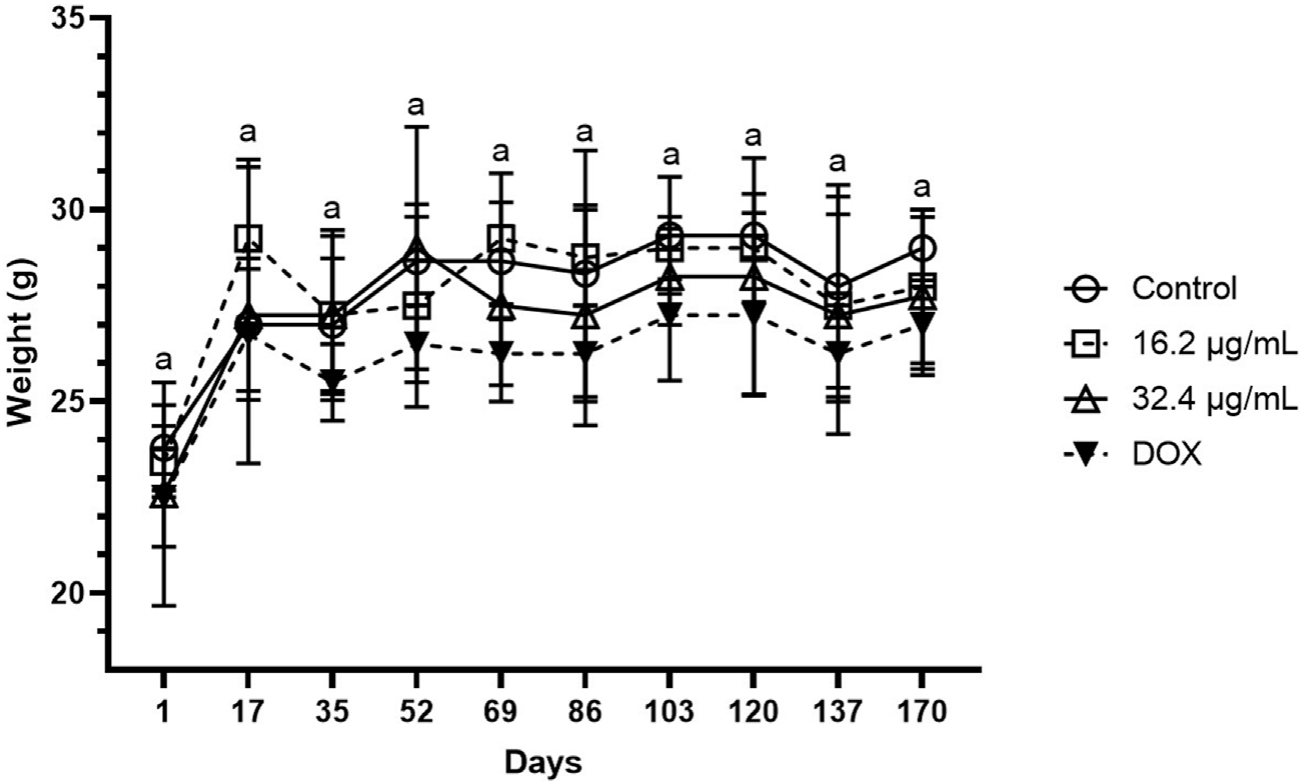
Monitoring of mice weight during a period of 170 days after tumor resection. During the monitoring period, there was no significant difference between groups. The results were analyzed using an ANOVA test; statistical significance (^a^ *p* < 0.05).

No presence of tumor recurrence was observed during the 170-day observation period after primary tumor resection in all treated mice (AgNPs-G and DOX). However, the control group without treatment showed a tumoral recurrence in the mammary zone starting at day 60. From that moment on, recurrence was observed in the rest of the mice at days 76 and 83 after primary tumor resection (Figure 5). No significant difference was found in mice weight during observation (Figure 6).

**FIGURE 7:**
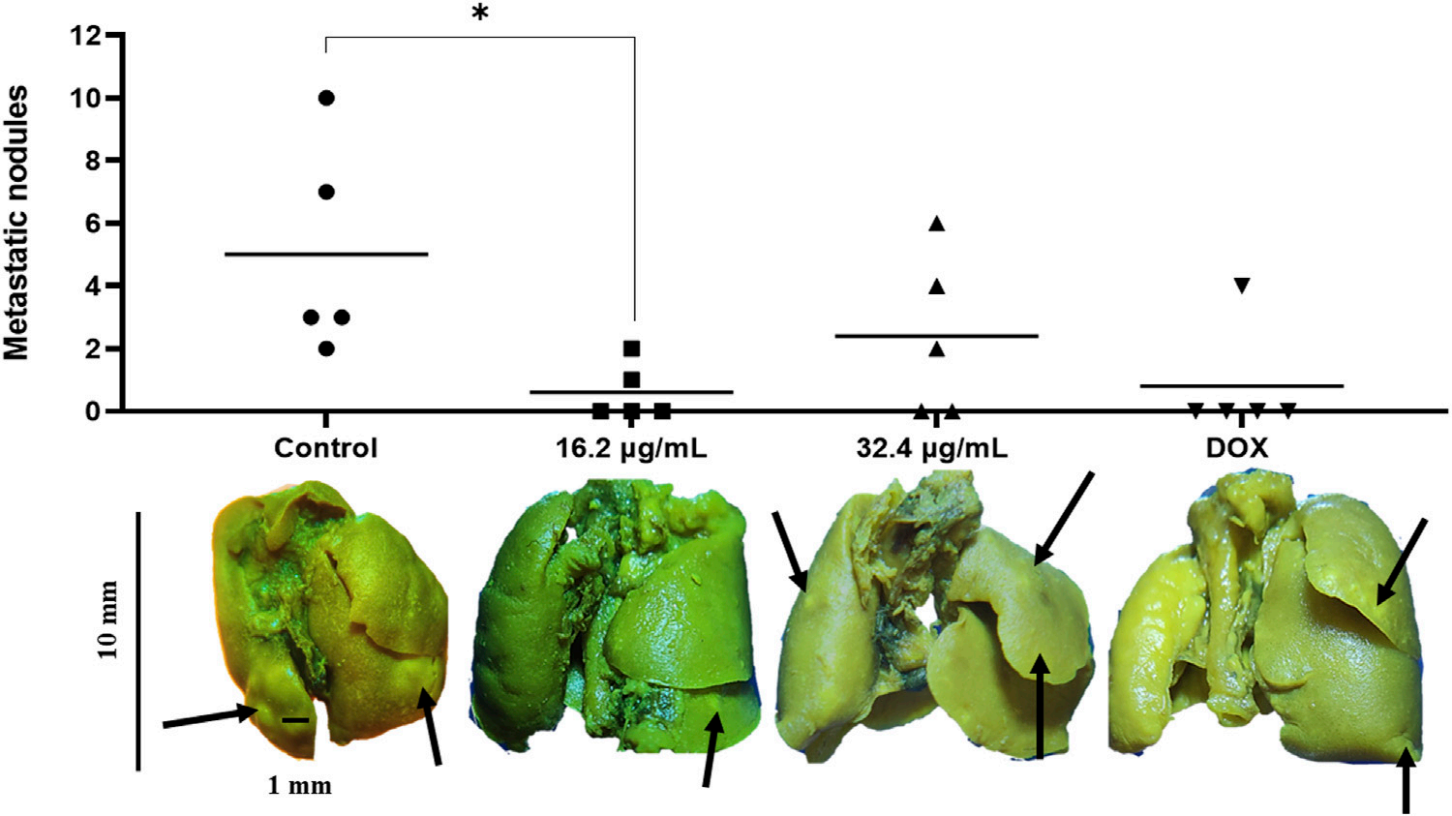
AgNPs-G administered neoadjuvantly reduce the appearance of metastatic nodules in the lungs. The control group exhibited a larger number of visible metastatic nodules in the lungs after tumor removal; however, only the group of AgNPs-G 16.2 μg/mL showed a significant difference. Mice from the control group were represented as individual dots; for 16.2 μg/mL, mice were represented as individual squared; for 32.4 μg/mL, mice were represented as individual triangle; finally, DOX mice were represented as upside down triangles. The results were analyzed using an ANOVA test; statistical significance (*p < 0.05).

After the observation period, it was observed that the control group showed the highest count of lung metastatic nodules among all groups. No significant difference was found (*p* < 0.05) between control without treatment and AgNPs-G 32.4 μg/mL and the DOX group with regard to the presence of metastatic nodule count. The group treated with AgNPs-G 16.2 μg/mL, however, reduced in a significant manner (*p* < 0.05) the presence of metastatic nodules when compared to the control group (Figure 7). However, the control group exhibited a larger nodule than all treatment groups.

### 3.5 AgNPs-G reduce tumor growth in metastatic lungs

**FIGURE 8:**
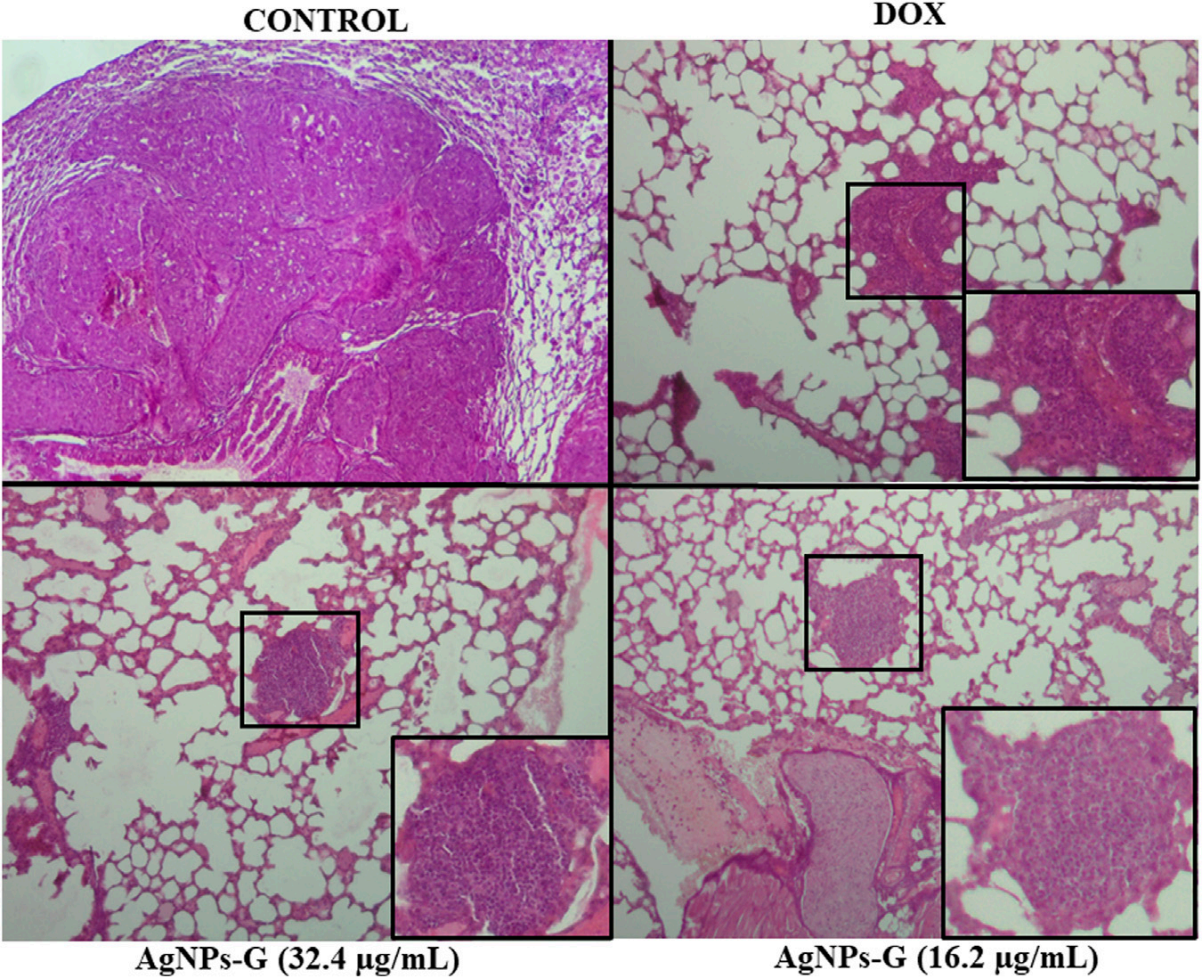
AgNPs-G administered neoadjuvantly reduce metastatic foci in the lungs. Representative images of H&E staining (40X), the control group showed the presence of tissue abnormalities alongside the loss of alveolar spaces. Treated groups showed the presence of metastatic foci bounded in a black square with a respective magnification.

The morphological evaluation of lung tissues across diverse experimental groups revealed discernible abnormalities consistent with metastatic foci of variable sizes. Within the negative control group, limited regions indicative of alveolar morphology were discerned, with most areas manifesting as metastatic nodules (Figure 8). Conversely, mice that were treated exhibited fewer abnormalities and diminished metastatic foci as visualized through hematoxylin and eosin (H&E) staining. Notably, mice treated with doxorubicin exhibited a reduction in the size of metastatic nodules, whereas those treated with AgNPs-G displayed the smallest metastatic nodules within the pulmonary tissue (Figure 8).

### 3.6 Collagen expressions are reduced in lungs after AgNPs-G treatment

**FIGURE 9:**
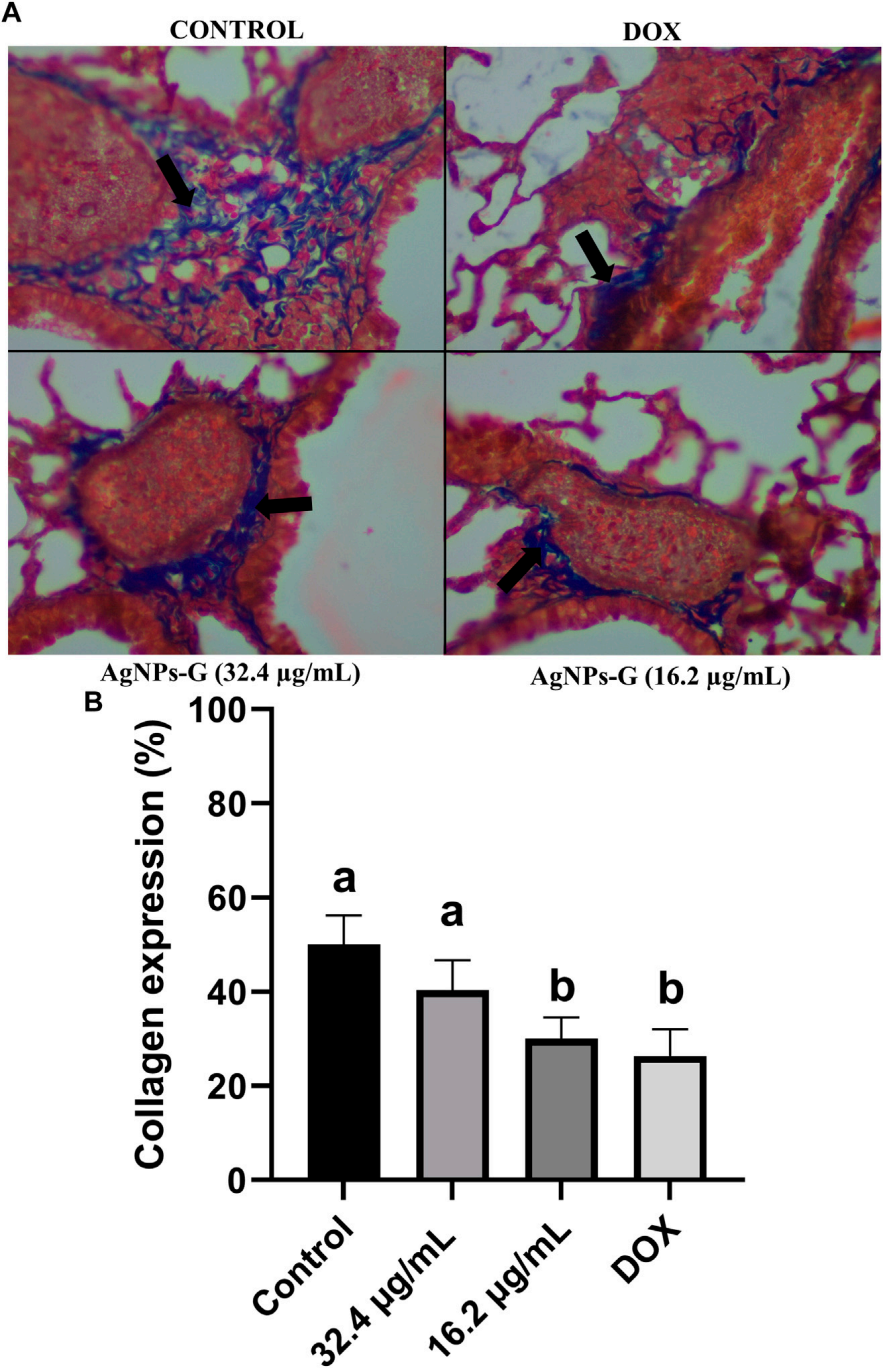
AgNPs-G reduce collagen presence around metastatic foci in the lungs after neoadjuvant administration. **(A)** Representative images of Masson’s staining reveal that more collagen fibers by blue coloration are accumulated surrounding metastatic nodules in the lungs. Black arrows indicate collagen presence. **(B)** Expression of collagen is only reduced with 16.2 μg/mL of AgNPs-G and DOX; statistical significance (^a,b^ *p* < 0.05).

In this study, Masson’s staining of lung sections was employed to discern the presence of collagen fibers, thereby establishing a correlation with tumor establishment and its associated extracellular matrix. The spatial distribution of metastatic foci was observed to be closely associated with regions exhibiting a higher concentration of collagen, both surrounding and in proximity to metastatic nodules (Figures 8, 9A).

Quantitative analysis revealed distinct patterns among the experimental groups. The negative control group exhibited the highest quantity of collagen fibers. Notably, treatment with AgNPs-G at a concentration of 32.4 μg/mL demonstrated no statistically significant difference compared to the control group. However, AgNPs-G at a concentration of 16.2 μg/mL and doxorubicin (positive control) exhibited a statistically significant difference in collagen fiber presence when compared to the control group, but not between these two (Figure 9B).

### 3.7 AgNPs-G reduce Ki67 expression in lungs

**FIGURE 10:**
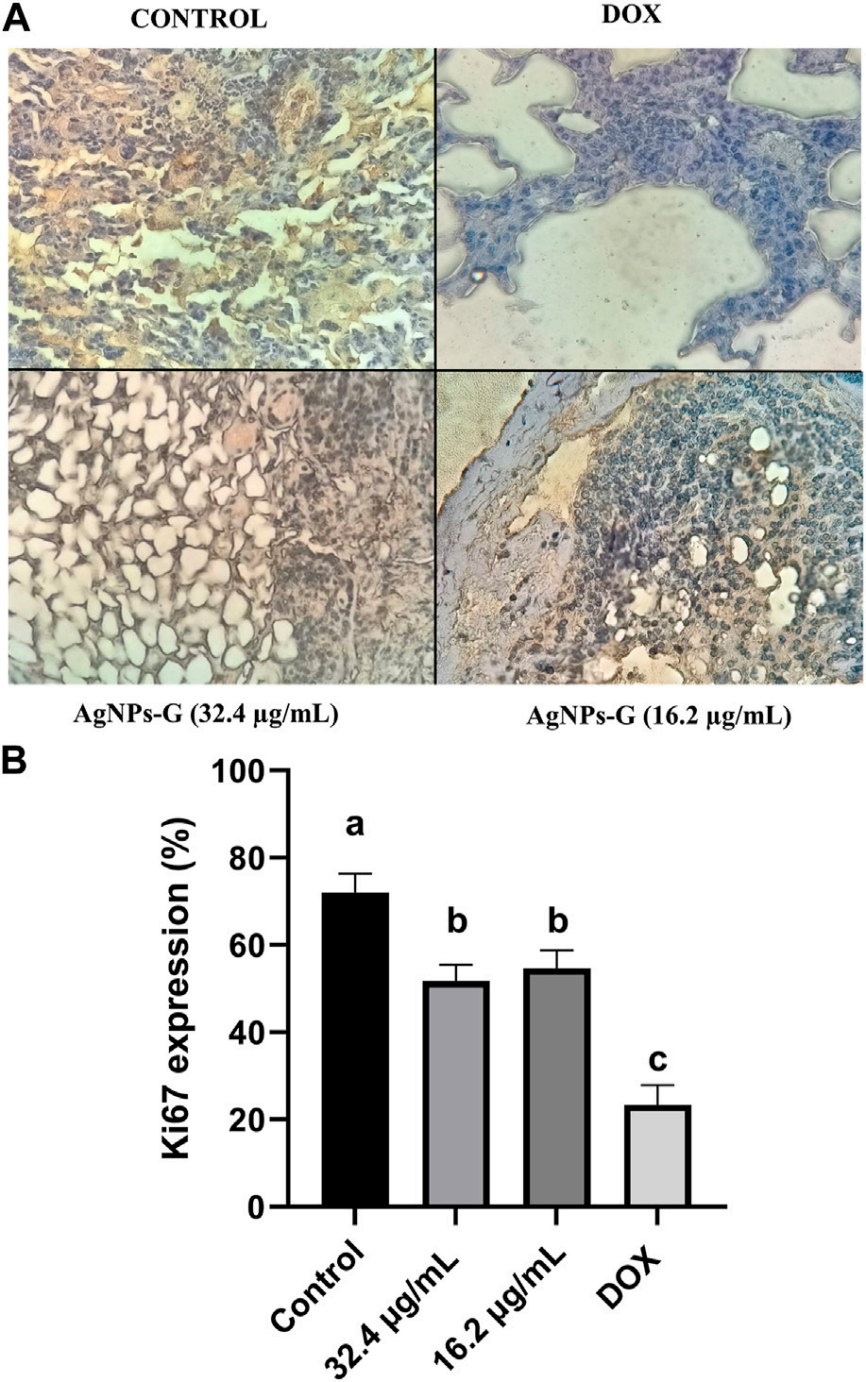
Ki67 expression is reduced in lung metastatic foci after neoadjuvant administration of AgNPs-G. **(A)** Representative images of immunohistochemistry reveal expression of Ki67 in the lung tissue. **(B)** Treated groups showed a reduction in Ki67 expression in all treated groups. The results were analyzed using an ANOVA test; statistical significance (^a,b^ *p* < 0.05).

The assessment of AgNPs-G influence on cell proliferation was conducted by evaluating Ki67 expression, revealing a significant reduction in treated mice (AgNPs-G at concentrations of 32.4 μg/mL or 16.2 μg/mL) compared to the control group (Figure 10). However, when compared with DOX treatment, the effect of AgNPs-G was minor (*p* < 0.05).

### 3.8 AgNPs-G induce mild damage to DNA from cancerous cells

**FIGURE 11:**
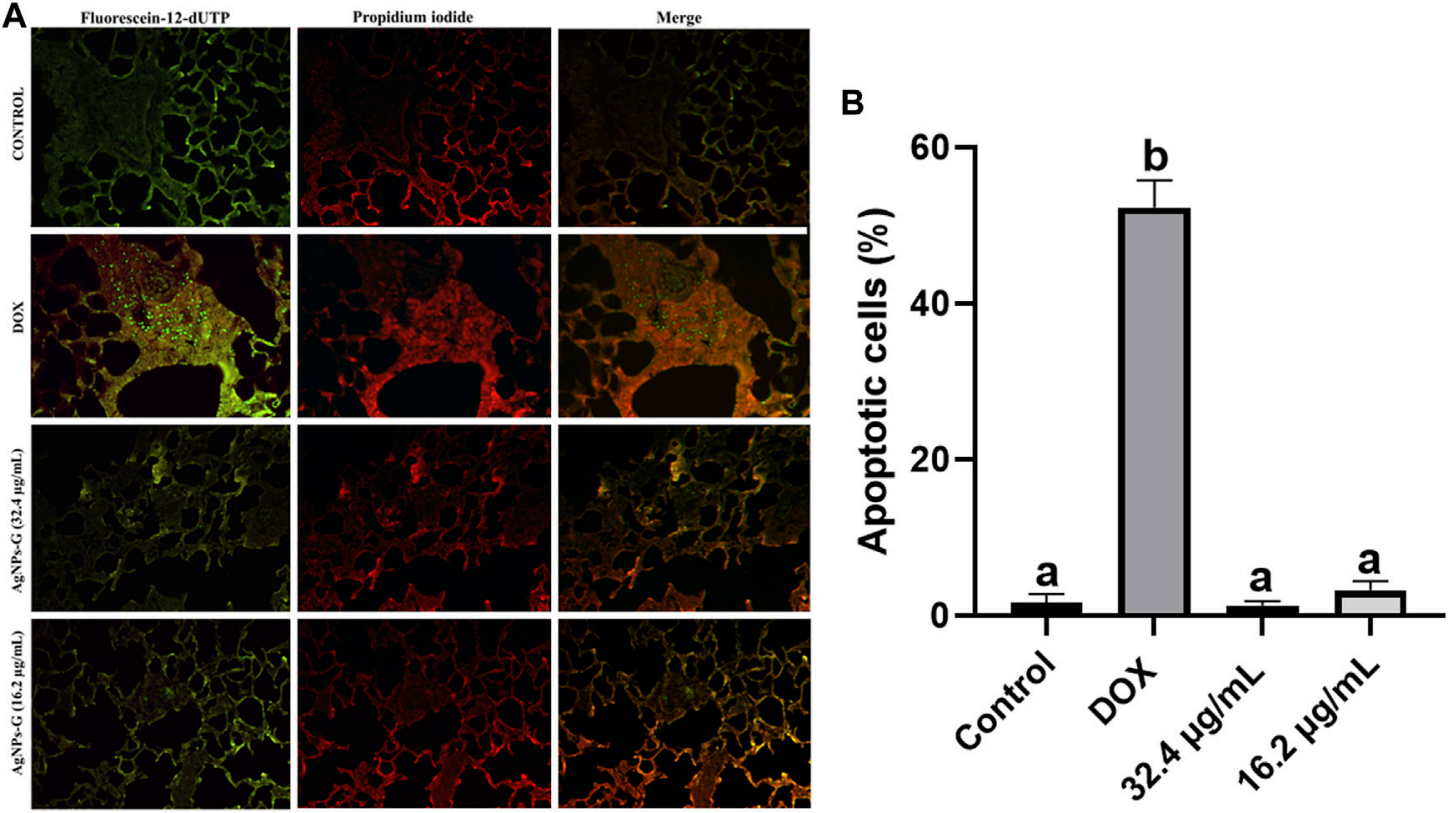
AgNPs-G apoptosis evaluation. **(A)** Representative images of the TUNEL assay reveal that our AgNPs-G when administrated neoadjuvantly does not induce apoptosis. **(B)** Groups treated with DOX showed an increase in apoptotic cells. The results were analyzed using an ANOVA test; statistical significance (^a,b^ *p* < 0.05).

Tumor lung sections from treated mice did not show significant difference (*p* < 0.05) in the percentage of apoptotic cells against control (Figure 11A). Only the DOX treatment increased the percentage of apoptotic cells when compared with the control (*p* < 0.05) (Figures 11A, B).

## 4 Discussion

Our results align with previous research findings, demonstrating the cytotoxic properties of silver nanoparticles (AgNPs) against breast cancer cells, as elucidated by Pucelik et al. (2022) and KumarBraj et al. (2015). Pucelik et al. (2022) employed a synthesis method involving sodium tetrahydroborate obtaining a particle size between 3 and 40 nm with a spherical form, achieving a remarkable 86% reduction in 4T1 cell viability using just 0.5 μg/mL of their AgNPs within a 24-h timeframe. Similarly, KumarBraj et al. (2015) employed AgNPs synthesis employing *Madhuca ionafolia* as a reducing agent with spherical shape and 12–50 nm size, resulting in a 50% decrease in 4T1 cell viability with 1.50 μg/mL dose over 24 h. This discrepancy in the results could be due to the different reducing agents used. In contrast to our AgNPs-G that require a higher concentration to induce a similar effect, there are some explanations for this: one is the form of our nanoparticles, presenting a quasi-spherical shape with a smaller average size of 5.991 nm, reported by our group of investigation (Felix et al., 2023). Another one is the amount of ions released, as has been demonstrated when using spherical AgNPs probing antibacterial effect in a shape-dependent manner, with a relation between the major surface area and antibacterial effect, as a sphere has major surface area than other morphologies; thus, more Ag ions are released (Cheon et al., 2019). The reducing agent was used, and cell type can be involved because the efficiency of nanomaterial endocytosis varies among different types of cancer cells. When employing glucose- and lactose-capped silver (Ag) nanoparticles, the uptake by L929 fibroblast cells increases the efficiency. Meanwhile, when treating A549 carcinoma cells, the lactose-capped silver nanoparticles exhibit a higher efficiency in endocytose than their glucose-modified counterparts (Sur et al., 2010).

The next question was whether our AgNPs-G could induce cytotoxic effect in an *ex vivo* experiment using homogeneous fragments of tumors in which a tumor environment exists that involve architecture and cellular diversity capable to protect malignant cells from treatment. The lethal doses 16.2 μg/mL of AgNPs-G during 24 h showed a higher index of cellular death. However, the study lacks measurements of the presence of nanoparticles inside tumor, and this remains to be assessed in future research because we evaluate this in an *in vivo* study. But we can suggest that in a media with AgNPs-G, these can be internalized probably due to their small size (5.991 nm) and as they induce tumor cellular death in an *ex vivo* study.

It is to know that an important pharmacologic feature that dictates systemic AgNP toxicity is the biodistribution and tissue accumulation of AgNPs following administration, and the distribution depends on the size, shape, and surface charge target organ (Blanco et al., 2018). We suppose that our AgNPs-G show attraction *in situ* toward the inside of breast cancer tumor mice. However, for a better comprehension of the mechanism of death tumor is necessary to carry out studies that should be consider in a posterior study to understand part of the mechanism of tumor toxicity and *ex vivo* in sliced tumor portions (Kim et al., 2023). The results of this experiment have provided compelling evidence for the intra-tumoral use of AgNPs-G *in vivo*, as confirmed by the substantial accumulation of silver within the tumor in an *in vivo* assay. However, the possibility of a targeted approach to avoid residual nonspecific accumulation should be considered. Notably, despite various authors discussing the potential of intra-tumoral AgNP administration, very few have directly investigated the presence of these nanoparticles within the tumor itself in their studies (Blanco et al., 2018; Ferdous and Nemmar, 2020). Also, as mentioned by Kovács et al. (2022), it is essential to gain a comprehensive understanding of how AgNPs affect the crosstalk between cancer cells and the various components of the tumor stroma upon their entry into the tumor microenvironment because the inherent biological characteristics of tumor cells significantly influence the efficacy of AgNPs. On the other hand, compared to i.t. administration, an i.v. administration of AgNPs-G, mainly accumulated in the spleen (0.91 ppm), kidney (2.15 ppm), and gallbladder (8.62 ppm), where highest concentrations were detected. The consequence of long-time exposure was not evaluated in this study, but it remains as a future perspective; the aim of this experiment was to demonstrate penetrability in different parts of the body. These results are consistent with others, where nanoparticles with a size up to 14 nm accumulate in the organs previously mentioned (Blanco et al., 2018; Ferdous and Nemmar, 2020). However, in gold nanoparticles *via* i.t. administration, a high concentration of gold in tumor tissue was reported (Li et al., 2016). Therefore, we decided to administer the AgNPs-G *via* i.t., to avoid accumulation of silver in different organs with a potential risk.

A neoadjuvant therapy must be performed to effectively reduce the tumor size before surgery (Selli and Sims, 2019). We found that our AgNPs-G administrated *via* i.t. can significantly reduce tumor volume in tumor-bearing mice to an average size of 100 mm^3^. Our results are consistent with other studies that report in tumor-bearing mice (induced with 4T1 cell line) that AgNPs can reduce tumors to an average size of 100 mm^3^ (Swanner et al., 2019). However, Brzóska et al. (2022) evaluated a synthesis of AgNPs reduced with citrate in mice bearing tumor from a 4T1 cell line, but no reduction in tumor volume was observed. This discrepancy in results could be attributed to factors such as size, shape, and coating that are known to alter the effect of nanoparticles (Auría et al., 2019).

In addition, we observed a decrease in the presence of metastatic nodules in mouse lungs that were subjected to neoadjuvant therapy with our AgNPs-G. One of the benefits of neoadjuvant therapy is the prevention or eradication of recurrence (McElnay and Lim, 2014). Brzoska et al. (2022) evaluated the ability to inhibit metastasis of 4T1 cells using citrate-reduced AgNPs and obtained an average count of around 11 metastatic nodules in the lung. Compared to the research carried out by Vo et al. (2015), where they observed up to 100 metastatic nodules in mice treated with chitosan immunotherapy and IL-12 neoadjuvant, our results demonstrated that the AgNPs-G showed better effect than other types of therapies such as immunotherapy, and furthermore avoided the recurrence in the zone where the primary tumor was surgically removed. Metastatic lesion count was reduced after AgNPs-G treatment when compared to the control group, followed by a reduction of collagen around metastatic nodules. The dominant component of the tumor extracellular matrix is fibrillar collagen, which compromises approximately 30% of its total protein (Myllyharju, 2004). Elevated collagen abundance affects the prognosis and progression of tumor as collagen content in cancerous cells leads to resistance to chemotherapy (Angre, et al., 2022). The reduction of collagen expression by our AgNPs-G treatment would be beneficial in a clinical setting due to reports of Kuczek et al. (2019), who found that a high collagen density negatively impacts the abundance of CD8^+^ cells in tumor and impairs cytotoxic activity in breast cancer tumors.

Ki67 is a proliferation marker, and cuantitative assessment provides an accurate estimate of the proliferation index of tumors. Our results indicate that AgNPs-G significantly reduced cancerous cell proliferation. High Ki67 index is associated with a greater risk of recurrence (Maolin et al., 2021), indicating that our AgNPs-G could serve as an effectively neoadjuvant treatment to reduce cancer recurrence. Nanoparticles have proven to be effective when implemented in cancer treatment. Mackenzie et al. demonstrated that nanoparticles utilized as a vector of Bcl-2 (a protein overexpressed in TNBC) inhibitor showed high efficacy in decreasing proliferation, which was denoted by reduced Ki67 staining.

The TUNEL assay serves as a useful method to indicate apoptosis. Our findings reveal that AgNPs-G does not induce apoptosis; nevertheless, the observed deficiency in the count of apoptotic cells may be attributed to the TUNEL assay as it was performed after 170 days of treatments; this was our observation period to evaluate recurrence. Other results showed that silver nanoparticles induce cell death by apoptosis (Ullah et al., 2020; Tao et al., 2021); nevertheless, these studies performed apoptosis assays from a primary tumor. However, it is not well understood how silver nanoparticles kill mammalian cells (Rohder et al., 2021). Further investigation is needed to understand how AgNPs-G induce cell death and the effect of silver residual from AgNPs-G on tumors.

This study is important in the ambit of discovery of alternatives to be used in pharmacological management and oncological surgery due to their ability to penetrate tumors, and their beneficial effects on tumor size and metastasis, which make them a promising tool for the treatment of cancer.

Furthermore, one of the limits of the study which should be considered more ahead is the lack of molecular studies associated to diminish the recurrence of breast cancer induced by AgNPs-G and is necessary to establish a comparative study with different synthesis and measurements of silver nanoparticles to determine its effectivity in this field of study.

In summary, our study provides valuable insights of the AgNPs-G potential for intra-tumoral and neoadjuvant cancer therapies. The unique characteristics of these nanoparticles, their ability to penetrate tumors, and their favorable impact on tumor size and metastasis make them a promising tool to treat cancer, accompanied with a reduction on Ki67 and collagen expression in metastatic foci. Further research is warranted to explore their full potential and optimize their use in clinical settings.

## Data Availability

The original contributions presented in the study are included in the article/Supplementary Materials; further inquiries can be directed to the corresponding authors.
